# Supplementary material for: Resolving the full spectrum of human genome variation using Linked-Reads
Source: Genome Res. 2019 Apr;29(4):635–45. doi: 10.1101/gr.234443.118 (PMC6442396; doi:10.1101/gr.234443.118)
Supplement: Supplemental Material [file supp_gr.234443.118_Supplemental_longranger-2.2.zip › longranger-2.2.tar.bz2!/lr2.2-public/lib/stan/cmdstan/stan/doc/api/html/var__stack_8hpp_source.html]

Stan: src/stan/agrad/rev/var\_stack.hpp Source File


|  |  |
| --- | --- |
|  | Stan  2.5.0  probability, sampling & optimization |


- Main Page
- Related Pages
- Namespaces
- Classes
- Files

- File List
- File Members

All Classes Namespaces Files Functions Variables Typedefs Enumerations Enumerator Friends Macros Pages

- src
- stan
- agrad
- rev

var\_stack.hpp

Go to the documentation of this file.

1 #ifndef STAN\_\_AGRAD\_\_REV\_\_VAR\_STACK\_HPP

2 #define STAN\_\_AGRAD\_\_REV\_\_VAR\_STACK\_HPP

3

4 #include <stdexcept>

5 #include <vector>

6 #include <stan/memory/stack\_alloc.hpp>

7

8 namespace stan {

9  namespace agrad {

10

11  // forward declaration of chainable

12  class chainable;

13  class chainable\_alloc;

14

15  extern std::vector<chainable\*> var\_stack\_;

16  extern std::vector<chainable\*> var\_nochain\_stack\_;

17  extern std::vector<chainable\_alloc\*> var\_alloc\_stack\_;

18  extern memory::stack\_alloc memalloc\_;

19

20  // nested positions

21  extern std::vector<size\_t> nested\_var\_stack\_sizes\_;

22  extern std::vector<size\_t> nested\_var\_nochain\_stack\_sizes\_;

23  extern std::vector<size\_t> nested\_var\_alloc\_stack\_starts\_;

24

31  class chainable\_alloc {

32  public:

33  chainable\_alloc() {

34  var\_alloc\_stack\_.push\_back(this);

35  }

36  virtual ~chainable\_alloc() { };

37  };

38

42  static inline bool empty\_nested() {

43  return nested\_var\_stack\_sizes\_.empty();

44  }

45

52  static inline void recover\_memory() {

53  if (!empty\_nested())

54  throw std::logic\_error("empty\_nested() must be true"

55  " before calling recover\_memory()");

56  var\_stack\_.clear();

57  var\_nochain\_stack\_.clear();

58  for (size\_t i = 0; i < var\_alloc\_stack\_.size(); i++)

59  delete var\_alloc\_stack\_[i];

60  var\_alloc\_stack\_.clear();

61  memalloc\_.recover\_all();

62  }

63

72  static inline void recover\_memory\_nested() {

73  if (empty\_nested())

74  throw std::logic\_error("empty\_nested() must be false"

75  " before calling recover\_memory\_nested()");

76

77  var\_stack\_.resize(nested\_var\_stack\_sizes\_.back());

78  nested\_var\_stack\_sizes\_.pop\_back();

79

80  var\_nochain\_stack\_.resize(nested\_var\_nochain\_stack\_sizes\_.back());

81  nested\_var\_nochain\_stack\_sizes\_.pop\_back();

82

83  for (size\_t i = nested\_var\_alloc\_stack\_starts\_.back();

84  i < var\_alloc\_stack\_.size();

85  ++i)

86  delete var\_alloc\_stack\_[i];

87  nested\_var\_alloc\_stack\_starts\_.pop\_back();

88

89  memalloc\_.recover\_nested();

90  }

91

96  static inline void start\_nested() {

97  nested\_var\_stack\_sizes\_.push\_back(var\_stack\_.size());

98  nested\_var\_nochain\_stack\_sizes\_.push\_back(var\_nochain\_stack\_.size());

99  nested\_var\_alloc\_stack\_starts\_.push\_back(var\_alloc\_stack\_.size());

100  memalloc\_.start\_nested();

101  }

102

103  static inline size\_t nested\_size() {

104  return var\_stack\_.size() - nested\_var\_stack\_sizes\_.back();

105  }

106

107  }

108 }

109 #endif

stan::agrad::recover\_memory

static void recover\_memory()

Recover memory used for all variables for reuse.

**Definition:** var\_stack.hpp:52

stan::agrad::start\_nested

static void start\_nested()

Record the current position so that recover\_memory\_nested() can find it.

**Definition:** var\_stack.hpp:96

stan::agrad::memalloc\_

memory::stack\_alloc memalloc\_

**Definition:** var\_stack.cpp:16

stan::agrad::nested\_size

static size\_t nested\_size()

**Definition:** var\_stack.hpp:103

stan::agrad::nested\_var\_stack\_sizes\_

std::vector< size\_t > nested\_var\_stack\_sizes\_

**Definition:** var\_stack.cpp:18

stan::agrad::chainable\_alloc

A chainable\_alloc is an object which is constructed and destructed normally but the memory lifespan i...

**Definition:** var\_stack.hpp:31

stan::agrad::chainable\_alloc::chainable\_alloc

chainable\_alloc()

**Definition:** var\_stack.hpp:33

stan::agrad::nested\_var\_alloc\_stack\_starts\_

std::vector< size\_t > nested\_var\_alloc\_stack\_starts\_

**Definition:** var\_stack.cpp:20

stan::agrad::var\_stack\_

std::vector< chainable \* > var\_stack\_

**Definition:** var\_stack.cpp:13

stan::agrad::chainable\_alloc::~chainable\_alloc

virtual ~chainable\_alloc()

**Definition:** var\_stack.hpp:36

stack\_alloc.hpp

stan::memory::stack\_alloc::start\_nested

void start\_nested()

Store current positions before doing nested operation so can recover back to start.

**Definition:** stack\_alloc.hpp:194

stan::agrad::var\_alloc\_stack\_

std::vector< chainable\_alloc \* > var\_alloc\_stack\_

**Definition:** var\_stack.cpp:15

stan::agrad::recover\_memory\_nested

static void recover\_memory\_nested()

Recover only the memory used for the top nested call.

**Definition:** var\_stack.hpp:72

stan::memory::stack\_alloc::recover\_nested

void recover\_nested()

recover memory back to the last start\_nested call.

**Definition:** stack\_alloc.hpp:203

stan::agrad::empty\_nested

static bool empty\_nested()

Return true if there is no nested autodiff being executed.

**Definition:** var\_stack.hpp:42

stan::agrad::nested\_var\_nochain\_stack\_sizes\_

std::vector< size\_t > nested\_var\_nochain\_stack\_sizes\_

**Definition:** var\_stack.cpp:19

stan::memory::stack\_alloc::recover\_all

void recover\_all()

Recover all the memory used by the stack allocator.

**Definition:** stack\_alloc.hpp:184

stan::agrad::var\_nochain\_stack\_

std::vector< chainable \* > var\_nochain\_stack\_

**Definition:** var\_stack.cpp:14


---

[ Stan Home Page ]

*© 2011–2014,
Stan Development Team.*
